# Dynamic Optical Coherence Elastography of the Anterior Eye: Understanding the Biomechanics of the Limbus

**DOI:** 10.1167/iovs.61.13.7

**Published:** 2020-11-03

**Authors:** Fernando Zvietcovich, Achuth Nair, Manmohan Singh, Salavat R. Aglyamov, Michael D. Twa, Kirill V. Larin

**Affiliations:** 1 Department of Biomedical Engineering, University of Houston, Houston, Texas, United States; 2 Department of Mechanical Engineering, University of Houston, Houston, Texas, United States; 3 College of Optometry, University of Houston, Houston, Texas, United States

**Keywords:** cornea, limbus, biomechanics, optical coherence elastography

## Abstract

**Purpose:**

Currently, the biomechanical properties of the corneo-scleral limbus when the eye-globe deforms are largely unknown. The purpose of this study is to evaluate changes in elasticity of the cornea, sclera, and limbus when subjected to different intraocular pressures (IOP) using wave-based optical coherence elastography (OCE). Special attention was given to the elasticity changes of the limbal region with respect to the elasticity variations in the neighboring corneal and scleral regions.

**Methods:**

Continuous harmonic elastic waves (800 Hz) were mechanically induced in the sclera near the corneo-sclera limbus of in situ porcine eye-globes (*n* = 8). Wave propagation was imaged using a phase-sensitive optical coherence tomography system (PhS-OCT). The eyes were subjected to five different IOP-levels (10, 15, 20, 30, and 40 mm Hg), and spatially distributed propagation velocities were calculated along corneal, limbal, and scleral regions. Finite element analysis (FEA) of the same regions under the same excitation conditions were conducted for further validation of results.

**Results:**

FEA demonstrated that the stiffness of the heterogeneous cornea-limbus-sclera transition can be characterized by phase velocity measurements of the elastic waves produced at 800 Hz in the anterior eye. Experimental results revealed that the wave speed in the limbus (*c_L_* = 6.5 m/s) is between the cornea (*c_c_* = 2.9 m/s) and sclera (*c_s_* = 10.0 m/s) at a physiological IOP level (15 mm Hg) and rapidly increases as the IOP level is increased, even surpassing the wave speed in the sclera. Finally, the change in elastic wave speed in the limbus (Δ*c_L_*∼18.5 m/s) was greater than in the cornea (Δ*c_c_* ∼12.6 m/s) and sclera (Δ*c_s_*∼8.1 m/s) for the same change in IOP.

**Conclusions:**

We demonstrated that wave-based OCE can be utilized to assess limbus biomechanical properties. Moreover, experimental evidence showed that the corneo-scleral limbus is highly nonlinear compared to the cornea and sclera when the eye-globe is deformed by an increase of IOP. This may suggest that the limbus has enough structural flexibility to stabilize anterior eye shape during IOP changes.

The cornea plays a critical role in vision because it is responsible for the majority of the total refracting power of the eye.^1^ Besides the transparency of the cornea, the geometry of its surface has a direct impact in the integrity of the eye's optics.^2^ Transparent corneal tissue transitions to the opaque sclera at a ring-shaped transitional border called the corneo-scleral limbus. These structures collectively form the outer tunic of the eye. Therefore, the preservation of vision quality is tied to the geometry and shape of the outer or fibrous tunic of the eye-globe.

The biomechanical properties of the outer tunic determine the structural characteristics of the ocular globe and may be altered in several diseased states, including axial elongation in myopia,^3^ pathological deformation in keratoconus,^4^ and iatrogenic keratoectasia following corneal refractive surgery.^5^ Studies demonstrated that the outer tunic is malleable, and that accommodation of the lens can produce changes in the corneal radius of curvature,^6^ shape, and size of the whole globe.^7^ Moreover, elevation of intraocular pressure (IOP) has the potential to produce deformations in the cornea; however, evidence has shown that IOP elevation does not produce disruptions in vision, suggesting the existence of a compensating mechanism that preserves visual acuity from global deformations.^8^ A number of studies have suggested that the corneo-scleral limbus performs this compensation.^9^^–^^12^

There is no consensus on the exact size, location, biomechanical properties, and role of the limbus. Numerical simulations conducted by Asejczyk-Widlicka et al.^10^ reported that, given the Young's moduli of cornea and sclera, an optimal limbal elasticity can be found to preserve vision quality when the eye is deformed by IOP variations. Other studies by Elsheikh et al.^12^ shows experimental evidence of the compensation role of limbus under the elevation of IOP in human eyes.

Optical coherence elastography (OCE) is an established field that allows for quantitative viscoelastic characterization of tissues with the micrometer-scale resolution.^13^^–^^16^ In particular, wave-based OCE, which leverages the propagation of mechanical waves for imaging mechanical contrast of soft tissues, has proven to be a useful technique for noninvasive elastography of the eye in a wide variety of applications.^17^ However, OCE of the eye has been mostly concentrated on the central cornea.^18^^,^^19^ OCE is more limited in the sclera due to its high rigidity (MPa order), which results in high propagation speeds and long wavelengths compared to the imaging field of view and diminished efficacy of wave tracking techniques.^20^^,^^21^ Finally, wave-based OCE of the limbal transitional region between the cornea and sclera presents a difficult challenge due to high mechanical heterogeneity causing back reflection and attenuation of waves, and the presence of smaller and stiffer features, such as the limbus, which has a radial thickness 1 to 2 mm.

In this work, we used OCE to image elastic (Lamb) waves excited at 800 Hz to characterize the elasticity of the cornea-limbus-sclera region in in situ porcine eye-globes (*n* = 8) at five different IOPs (10, 15, 20, 30, and 40 mm Hg). Two-dimensional spatially distributed speed maps were calculated for the corneal, limbus, and scleral regions. The change of elasticity at the limbus was analyzed with respect to IOP, and the elasticity of the cornea and sclera. Finally, finite elements analyses (FEAs) were conducted to validate the capabilities of Lamb wave propagation at 800 Hz in detecting elasticity changes along the heterogenous shell-shaped anterior eye segment. Special emphasis is given in investigating the relationship between elastic (Lamb) wave speed and Young's modulus in the limbus given its reduced lateral extent compared to the corneal and scleral regions.

## Methods

### Sample Preparation and Experiment Design

**Figure 1.**
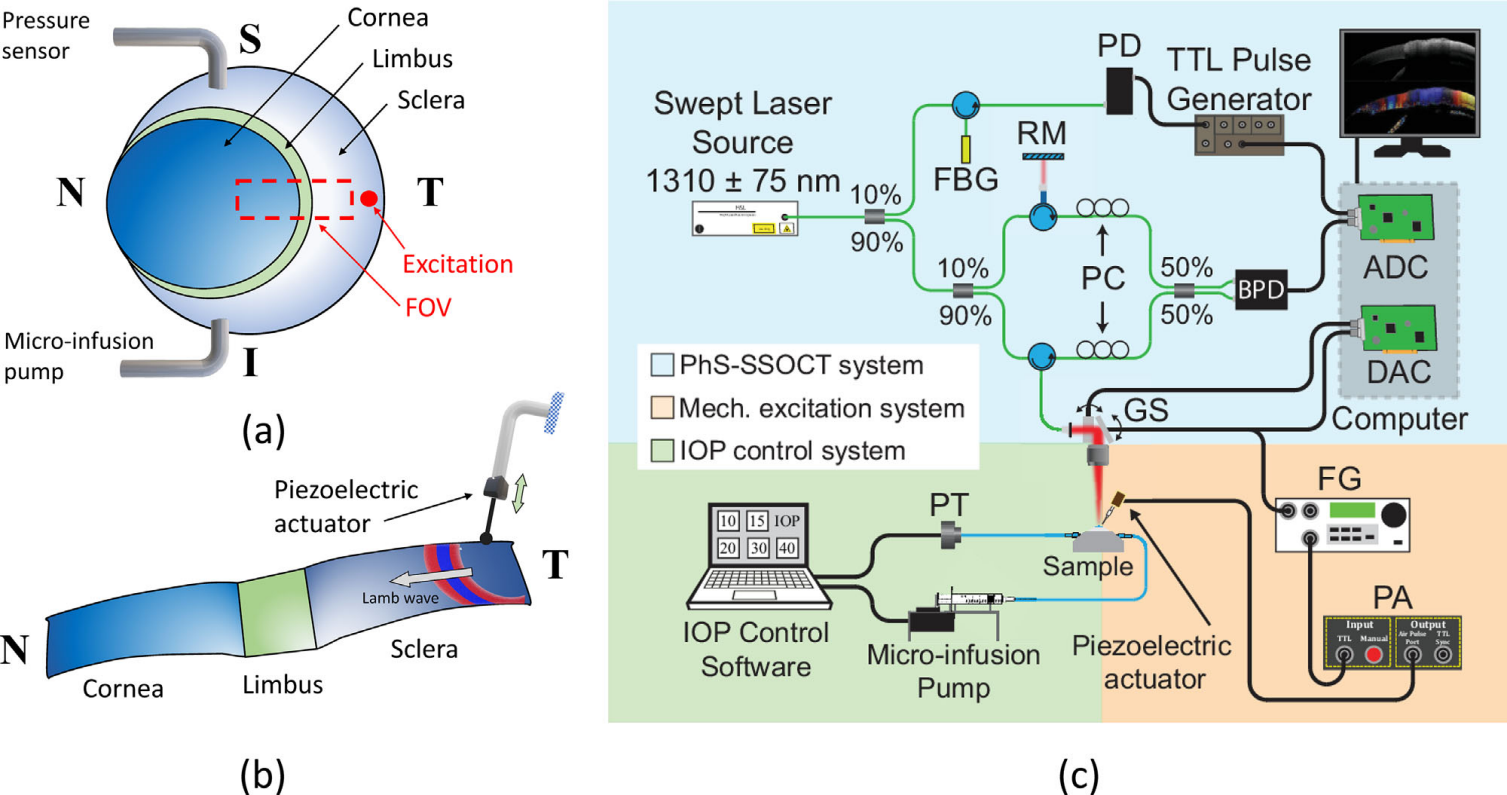
Experimental setup and sample preparation. (**a**) Three dimensional schematic of the scanning field-of-view (FOV) in an ex vivo porcine eye cannulated with two needles for artificial IOP control. N, nasal; T, temporal; S, superior; I, inferior. (**b**) Two dimensional schematic of cornea-limbus-sclera interface and excitation method using a piezoelectric actuator. (**c**) Experimental optical coherence elastography setup. FBG, Fiber-Bragg grating; RM, reference mirror; PC, polarization controllers; PD, photodetector; BPD, balanced photodetector; ADC, analog to digital converter; DAC, digital to analog converter; GS, Galvo scanners; PT, pressure transducer; FG, function generator; PA, power amplifier.

Porcine eyes (*n* = 8) were obtained (Sioux-Preme Packing Co., Sioux City, IA, USA), and extraneous tissues, such as muscles and fat, were removed. Experiments were conducted within 48 hours of eye enucleation. Eyes were placed in a holder, and oriented for imaging of the cornea-limbus-sclera region along the nasal-temporal direction and cannulated with two needles for artificial IOP control along the superior-inferior meridian (see Fig. 1a). The first needle port was connected to a micro-infusion pump, whereas the second needle was connected to a pressure gauge for the closed-loop IOP control using a previously reported system.^22^

For the excitation of mechanical waves in the anterior segment, a solid metal probe with a rounded spherical tip (diameter ∼ 1 mm) was positioned in the scleral region ∼ 12 mm away from the corneal-sclera border along the nasal-temporal direction. The tip was slightly touching the scleral tissue and was aligned normal with respect to the scleral surface (see Fig. 1b).

### Experimental Setup

The OCE acquisition system is the combination of a phase-stabilized swept-source optical coherence tomography (PhS-SSOCT) system^23^ with a mechanical piezoelectric-based excitation system. The PhS-SSOCT system was composed of a swept laser source (HSL2000; Santec Inc., Hackensack, NJ, USA) with a central wavelength of ∼ 1310 nm, bandwidth of ∼ 130 nm, and output power of ∼ 39 mW. The axial and lateral resolution of the OCT sample beam was ∼ 11 µm and ∼ 16 µm, respectively, in air. The A-line acquisition rate was 30 kHz (temporal resolution Δ*t* ∼ 33.3 µs). The axial sampling resolution of the system was ∼ 4 µm per pixel in tissue. The displacement stability was measured as ∼ 40 nm in these experiments.

The mechanical excitation system was composed of piezoelectric actuator of 3 × 3 mm cross section and 5 mm length (SA030305; PiezoDrive, Shortland, Australia) restrained by a stabilization arm on one side and connected to the metal probe on the other side (see Fig. 1b). A train of 8 cycles of an 800 Hz sinusoidal signal was output by a function generator (DG4062; Rigol Technologies, Beijing, China), amplified by a miniature piezo amplifier (PDu100B; PiezoDrive), and delivered to the piezoelectric transducer for the generation of mechanical waves in the tissue. An excitation frequency of 800 Hz was chosen to achieve enough mechanical resolution to distinguish elasticity gradients along the surface of the sample according to Kirby et al.^13^ The mechanical resolution in this study refers to the accuracy of the imaging method in detecting speed changes along space. Even though higher frequencies are desired to improve the mechanical resolution,^13^ they produced stronger wave attenuation impeding the elastography of the complete cornea-limbus-sclera section.

### OCE Measurements

The M-B-mode protocol was used for the acquisition of motion in samples in 2D along the *xz*-plane, where the *x*-axis and *z*-axis correspond to lateral and axial (depth) directions, respectively.^24^ This protocol consists of the OCT acquisition of M = 601 A-line repetitions (∼ 20 ms) for a given lateral position, whereas the excitation system is triggered to produce mechanical waves in the sample. Subsequently, the steering mirror controlled by a galvanometer (GVS002; Thorlabs Inc., Newton, NJ, USA) moves the OCT beam to the next consecutive lateral position for the next acquisition of M A-lines. A lateral distance of ∼ 15.7 mm was imaged using 500 *x*-samples (lateral sampling resolution of Δx∼31.5 µm). The data was reorganized in 2D + time format resulting in an equivalent frame rate of 30 kHz. The piezo excitation was synchronized with the OCT system frame trigger, as described previously.^25^

Particle velocity (rate of axial motion) was calculated from the depth-dependent phase φ(*x*_0_, *z*, *t*) difference of two consecutive complex-value A-lines for a given *x*_0_-position as^26^:

(1) $$v_{z}\left(x_{0},\;\;z,\;\;t\right)=\Delta \phi \left(x_{0},z,t\right)\frac{\lambda_{0}}{4\pi n\Delta t\;},$$

where Δφ(*x*_0_,  *z*,   *t*) = φ(*x*_0_, *z*, *t* + Δ*t*) − φ(*x*_0_, *z*, *t*), λ_0_ was the central wavelength of the OCT system, *n* is the refractive index of the sample (*n* ∼ 1.376 for cornea,^27^ and *n* ∼ 1.41 for sclera^28^), and Δ*t* was the temporal resolution. Then, 2D spatial-dependent particle velocity *v_z_* (*x*,  *z*,   *t*) is generated after applying Equation 1 to every lateral position. Refractive index mismatch between air and tissue was compensated using the approach described by Song et al.^29^

For each eye, OCE measurements of mechanical wave propagation in the cornea-limbus-sclera region were conducted at five different IOPs (10, 15, 20, 30, and 40 mm Hg). In between measurements, the eye was hydrated using a 1X PBS solution.

### Phase Velocity Estimation

As previously reported by our and other research groups,^17^^,^^30^^,^^31^ mechanical waves propagating in the cornea and sclera are Lamb waves due to the thin-shell boundary type of such tissues (thicknesses ranging from 0.4–2 mm) compared to the excitation wavelength. Lamb waves are a dispersive and have been modeled by Han et al.^30^^,^^32^ using the modified Rayleigh-Lamb frequency equation (mRLFE) accounting for air and aqueous humor interfaces at the upper and lower surfaces of the cornea/sclera tissues, respectively. Lamb wave propagation is guided by the surfaces of tissues; therefore, a spatial 2D phase velocity estimation method accounting for arbitrary propagation directions was selected. Compensation of speed due to the curvature of tissues with respect to the OCT measurement axis was taken into account as proposed by Han et al.^33^

The 2D phase-derivative method^34^^,^^35^ leveraging the complex-valued spatial particle velocity field *v_z_*^*Cplx*^(*x*, *z*) was used for the local phase velocity estimation of the Lamb waves. At a particular spatial location, *v_z_*^*Cplx*^(*x*, *z*) is obtained by (1) calculating the Fourier transform along the temporal dimension resulting in *V_z_*(*x*, *z*; ω), and (2) evaluating the transformed signal at the excitation frequency ω_0_ = 2π · 800 rad/s: *v_z_*^*Cplx*^(*x*, *z*) = *V_z_*(*x*, *z*; ω_0_). Then, the complex-valued particle velocity can be expressed in terms of magnitude, *A*(*x*, *z*), and phase, θ(*x*, *z*), as *v_z_*^*Cplx*^(*x*, *z*) = *A*(*x*, *z*)*e*^*i*θ(*x*, *z*)^. Within a small region of interest (ROI) of L × L mm^2^ centered at (*x*_0_, *z*_0_), orthogonal wave numbers corresponding the propagation directions *x* and *z,* can be calculated base of the spatial derivate of phase as:

(2) $$k_{x}\left(x_{0},z_{0}\right)=\frac{\partial \theta \left(x,\;z_{0}\right)}{\partial x},$$

and

(3) $$k_{z}\left(x_{0},z_{0}\right)=\frac{\partial \theta \left(x_{0},\;z\right)}{\partial z},$$

respectively. Finally, the equivalent 2D spatial Lamb wave speed map is found using:

(4) $$c_{2D}=\frac{\omega_{0}}{\sqrt{{k_{x}}^{2}+{k_{y}}^{2}}}.$$

### Finite Element Analysis

**Figure 2.**
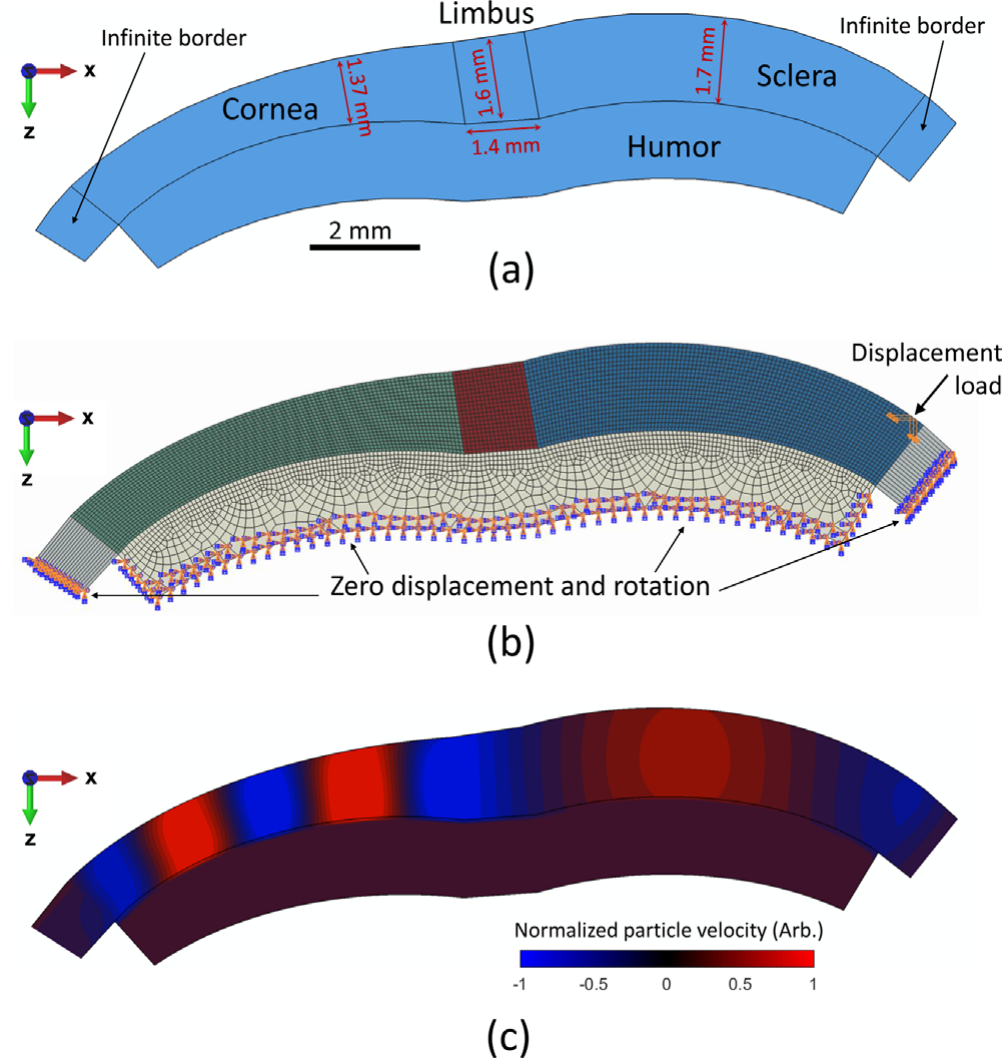
Finite element simulation of wave propagation in the anterior eye segment. (**a**) Schematic of the 2D finite element model showing geometry, borders, and dimensions. (**b**) Finite element mesh, boundary conditions, and loading position. (**c**) Particle velocity field along depth (*z*-axis) obtained after FEM at instant *t*_0_ = 7 ms for the study case A (*E_L_* = 400 kPa, *E_s_* = 2000 kPa), when *E_c_* = 22 kPa. Color bar represents normalized particle velocity in arbitrary units along the z-axis. Wave speed was calculated along the path of propagation defined by the surface of the medium.

Numerical simulations of Lamb wave propagation in the anterior eye segment were conducted using FEA in Abaqus/CAE version 2019 (Dassault Systems, Velizy-Villacoublay, France). A 2D geometry representing the cornea-limbus-sclera transition was created taking into consideration previous reported research,^10^^,^^36^ histological images,^37^ and structural OCT images^38^ for each tissue type in porcine eyes, as shown in Figure 2a. We used plane strain and linear elements connecting at the bottom tissue boundary with liquid representing the aqueous humor. Infinite plane strain elements were used at the outward lateral boundaries of the cornea and sclera to avoid strong wave reflections and standing waves.

The solid model was meshed using a grid size of ∼ 80 µm and linear hexahedral elements within the cornea-limbus-sclera section. A total of ∼ 130,000 elements were generated. A zero displacement and rotation boundary condition was applied in the bottom layer of the aqueous humor and extreme lateral boundaries of the infinite borders. In order to replicate the mechanical excitation produced by the probe tip touching the tissue, a temporally transient (8 cycles of a 800 Hz signal) and normally oriented displacement load was located at the surface of the scleral region, as shown in Figure 2b. The type of simulation was selected to be dynamic-explicit with a minimum temporal discretization of 1 µs and analyzed during a time range of 10 ms.

Linear viscoelastic material properties were assigned to the cornea, limbus, and sclera assuming a material density of ρ = 988 kg/m^3^, a Poisson's ratio of *v* =  0.498 (nearly incompressible), and a shear viscosity η = 0.55 Pa · s (averaged from measurements reported in Han et al.^30^). In liquid, a density of δ_*A*_ = 1000 kg/m^3^ and bulk modulus *K* = 2.2 GPa were selected.^39^ We conducted three study cases by varying the Young's modulus (*E*) of each section:

▪ Study A: Invariant limbus (*E_L_* = 400 kPa) and sclera (*E_s_* = 2000 kPa) elasticity; variable cornea elasticity (*E_c_* = [22, 50, 100, 200, 300, 500, 1000, and 2000] kPa). A total of eight simulation cases.
▪ Study B: Invariant cornea (*E_c_* = 50 kPa) and sclera (*E_s_* = 2000 kPa) elasticity; variable limbus elasticity (*E_L_* = [50, 100, 300, 700, 1200, and 2000] kPa). A total of six simulation cases.
▪ Study C: Invariant cornea (*E_c_* = 22 kPa) and limbus (*E_L_* = 400 kPa) elasticity; variable sclera elasticity (*E_s_* = [100, 200, 800, 2000, and 4000] kPa). A total of five simulation cases.

For every simulation case, particle velocity along the depth axis (*z*-axis) was stored and processed using the phase-derivative method. Figure 2c shows the 2D normalized particle velocity map at instant *t*_0_ = 7 ms produced by the propagation of Lamb waves from the sclera to corneal regions for study A case *E_c_* = 22 kPa, *E_L_* = 400 kPa, and *E_s_* = 2000 kPa.

## Results

### Wave Propagation in the Anterior Eye Segment Using Finite Elements Analysis

**Figure 3.**
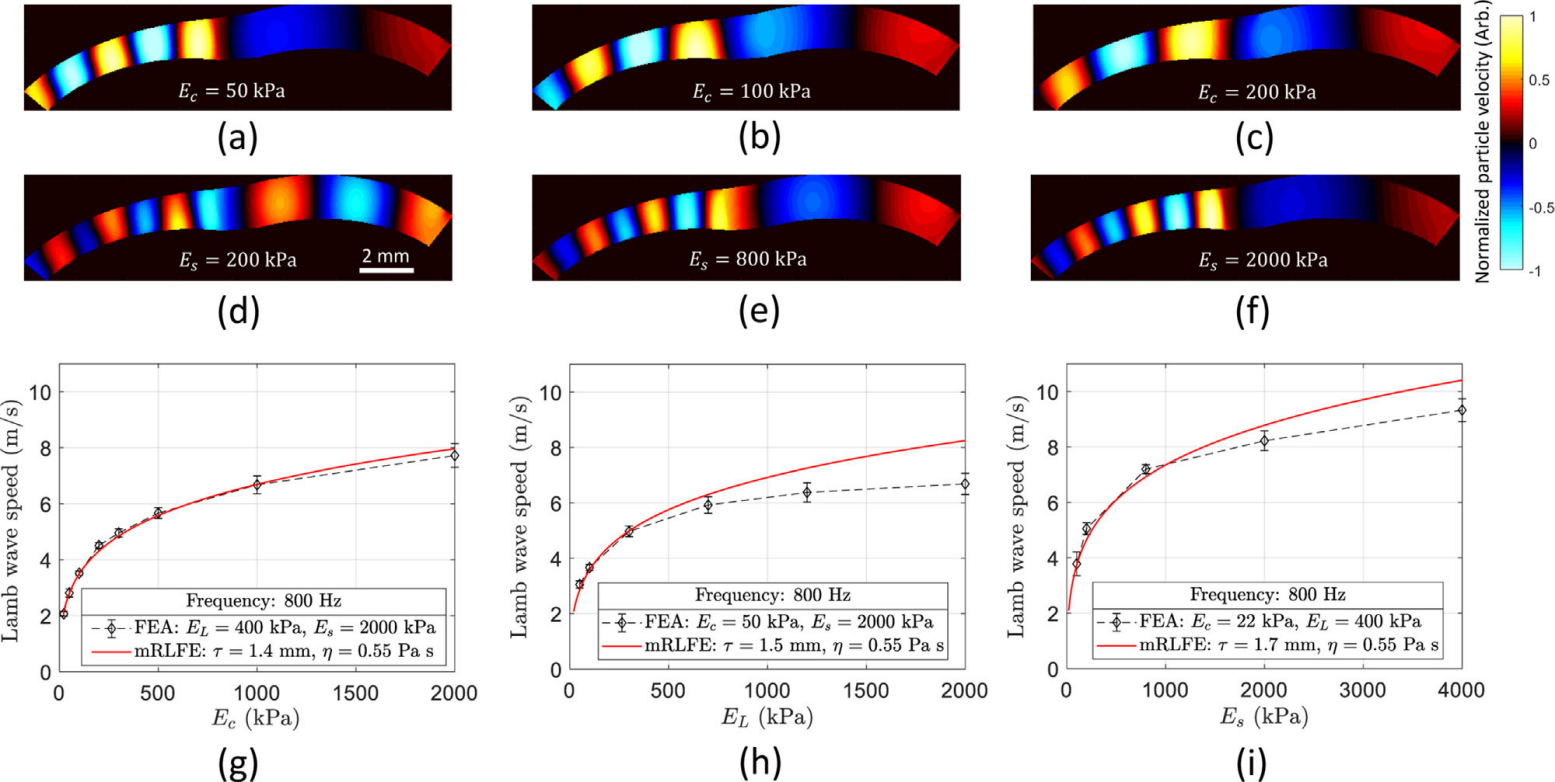
Numerical simulation results of wave propagation in the anterior eye segment. (**a–c**) FEA particle velocity fields for study A in cases *E_c_* = 50 kPa **a**, *E_c_* = 100 kPa **b**, and *E_c_* = 200 kPa **c**. (**d–f**) FEA particle velocity fields for study C in cases *E_s_* = 200 kPa **d**, *E_s_* = 800 kPa **e**, and *E_s_* = 2000 kPa **f**. Color bar represents normalized particle velocity along depth axis in arbitrary units. (**g–i**) Lamb wave speed versus Young's modulus plots for study cases: A – variable *E_c_* – **g**, B – variable *E_L_* – **h**, and C – variable *E_s_* – **i**. Error bars denote variability of speed in each region. Modified Rayleigh-Lamb frequency equation was fitted in all cases for excitation frequency = 800 Hz (*red plot*).

Figures 3a to 3c show 2D normalized particle velocity snapshots extracted at time *t*_0_ = 7 ms corresponding to study C for cases: *E_s_* = 200 kPa, *E_s_* = 800 kPa, and *E_s_* = 2000 kPa. Here, Lamb wavelengths get larger in the sclera region as *E_s_* increases. Similarly, for study A, wavelengths get larger in the cornea region as its Young's modulus increases in cases *E_c_* = 50 kPa (Fig. 3d), *E_c_* = 100 kPa (Fig. 3e), and *E_c_* = 200 kPa (Fig. 3f).

For each study case (A, B, and C), the phase-derivative method was used to estimate phase speed at the cornea (Fig. 3g), limbus (Fig. 3h), and sclera (Fig. 3i) regions versus the Young's moduli set in the simulation, respectively. The relationship between Young's modulus and speed is not quadratic, as expected in shear waves in uniform, homogeneous, and infinite media. On the contrary, this relationship obeys the modified Rayleigh-Lamb wave equation^30^ for the same set of parameters defined in each simulation case and average thicknesses τ = 1.4 mm (cornea), τ = 1.5 mm (limbus), and τ = 1.7 mm (sclera; as shown in Figs. 3g–i).

**Figure 4.**
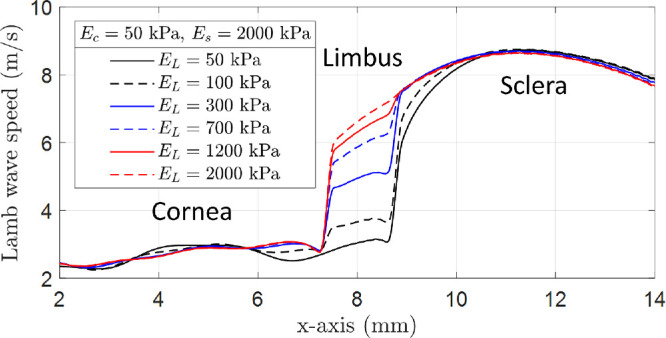
Lateral-dependent Lamb wave speed obtained from finite element simulations in the study case B: variable Young's modulus in the limbal region. Lamb wave speed at limbus increased as its Young's modulus was increased in the simulation. On the contrary, wave speed in the cornea and sclera did not change as its Young's moduli were kept constant: E_c_ = 50 kPa, E_s_ = 2000 kPa.

Finally, Figure 4 shows a lateral-dependent Lamb wave speed for study B in which the speed estimations in the cornea and sclera regions are approximately constant and the speed in the limbus region increases as its Young's modulus is increased. This demonstrates that speed variation in the limbus region can be detected using an 800 Hz propagating wave and is related to changes in limbal elasticity even when located at the boundary of two media with very different elastic properties and considering its small lateral size (∼ 1.4 mm) compared to the excitation wavelength.

### OCE Measurements of Cornea-Limbus-Sclera Lamb Wave Speed Versus IOP

**Figure 5.**
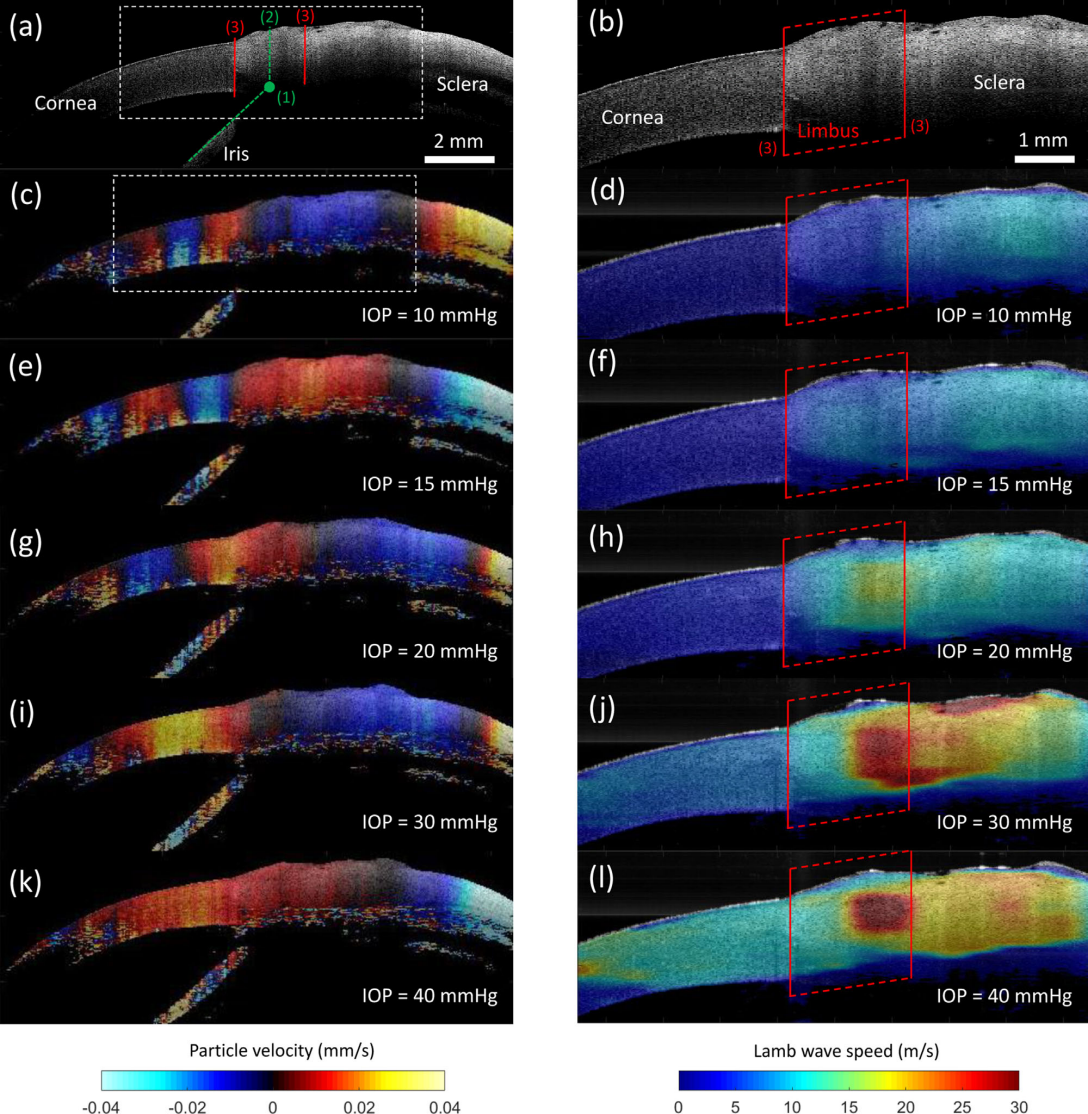
Experimental simulation results of wave propagation in the anterior eye segment. (**a,** **b**) B-mode structural images of the *ex vivo* porcine anterior eye segment in the OCT **a** and OCE **b** field-of-view. The delimitation process of limbus is indicated in **a** in three steps. As a result, the limbus region is segmented laterally in **b** by using two parallel lines. The OCE field-of-view is represented as a dashed box in **a**. Cornea, sclera, and limbus are labeled in **b**. (**c, e, g, i, k**) Wave propagation captured in the OCT field-of-view for IOPs: 10 mm Hg **c**, 15 mm Hg **e**, 20 mm Hg **g**, 30 mm Hg **i**, and 40 mm Hg **k**. Color bar represents particle velocity *v_z_*(*x*, *z*) along the *z*-axis in mm/s. (**d, f, h, j, l**) Two dimensionial Lamb wave speed maps calculated in the OCE field-of-view for IOP levels: 10 mm Hg **d**, 15 mm Hg **f**, 20 mm Hg **h**, 30 mm Hg **j**, and 40 mm Hg **l**. Color bar represents Lamb wave speed in m/s.

Spatial 2D Lamb wave speed was calculated at each porcine eye (*n* = 8 eyes) when subjected to 10, 15, 20, 30, and 40 mm Hg IOPs. Figure 5a shows the B-mode structural image of the OCT field-of-view where wave propagation was acquired. The iris, cornea, and sclera can be easily identified and are labeled. Figure 5b shows the B-mode image of the OCE field-of-view where estimations of Lamb wave speed were conducted and corresponds to the dashed box in Figure 5a. Here, the limbus region was selected based on previous reports using OCT imaging and histology^40^ and giving extra tolerance in the segmentation as follows: (1) we define a starting point as the intersection of the projective line following the iris with any visible posterior surface in the eye's anterior segment, as shown in Figure 5a; then (2) from that point we trace a line perpendicular and toward the surface of the eye's anterior segment; finally, (3) we roughly delimit the lateral boundaries of limbus by tracing parallel segments separated 1 mm to the left and right side of the line calculated in (2).

Particle velocity 2D snapshots (motion frames extracted at time *t*_0_ = 1 ms) are shown in Figures 5c, 5e, 5g, 5i, and 5k for IOPs of 10, 15, 20, 30, and 40 mm Hg, respectively. For IOP = 10 mm Hg, the Lamb wave wavelength was smaller in the cornea compared to the sclera, as predicted by FEA. As IOP increased, wavelengths became longer in both regions, indicating an increase of rigidity of both tissues. Figures 5d, 5f, 5h, 5j, and 5l show Lamb wave speed maps when the eye-globe IOP was at 10, 15, 20, 30, and 40 mm Hg, respectively. The speed maps confirm the nonlinear biomechanical properties of the cornea and sclera when subjected to increasing strains induced by IOP. Interestingly, Lamb wave speed maps reveal more information about the change of rigidity in the limbus region that is not evident in the particle velocity snapshots. In particular, a spike in limbus speed was detected at higher IOPs.

**Figure 6.**
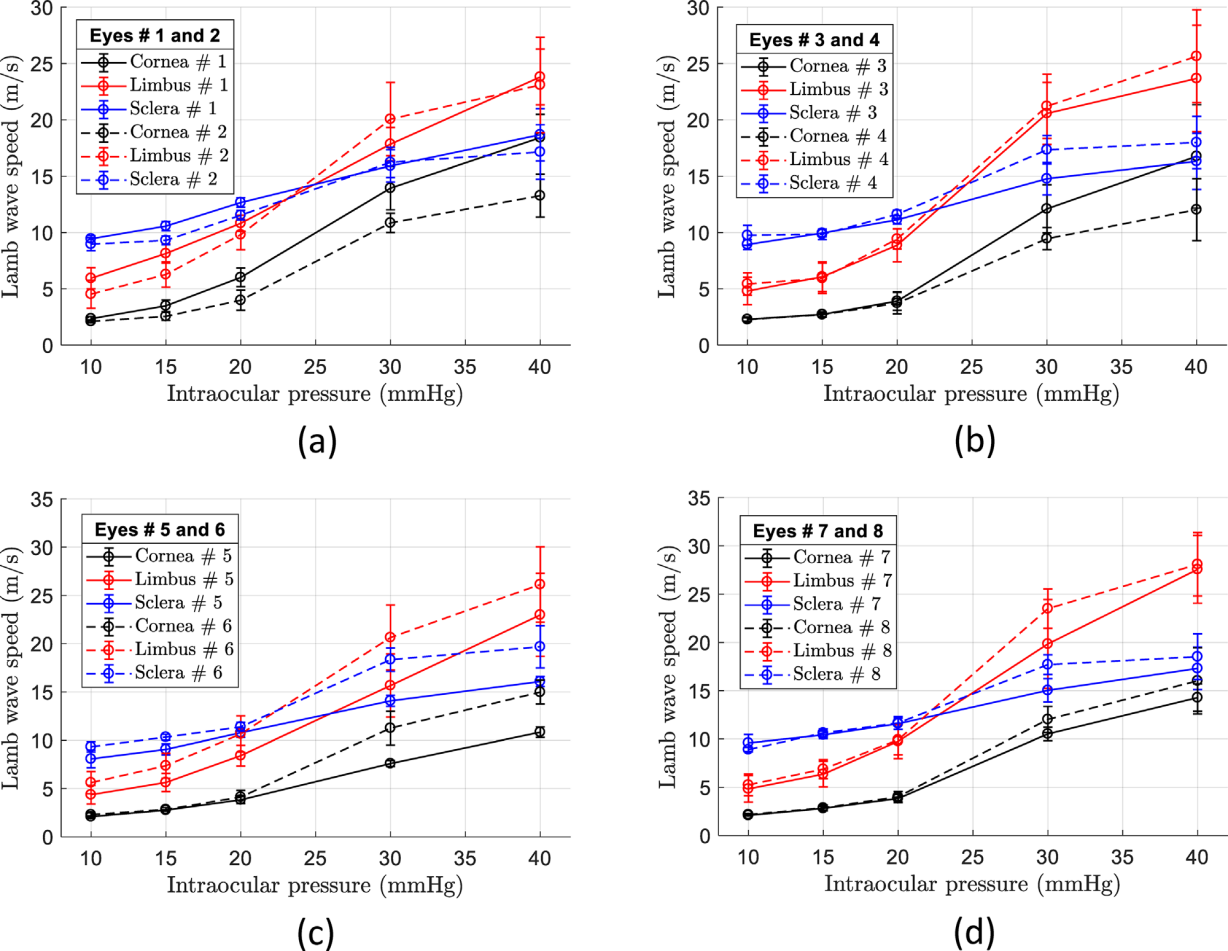
Lamb wave speed analysis in individual samples. Lamb wave speed versus IOP plots for each imaged region (cornea, limbus, and sclera) regions of in situ porcine eye samples #1, 2 (**a**), #3, 4 (**b**), #5, 6 (**c**), and #7, 8 (**d**). Data points and error bars represent mean and standard deviation of speed within each region, respectively.

**Figure 7.**
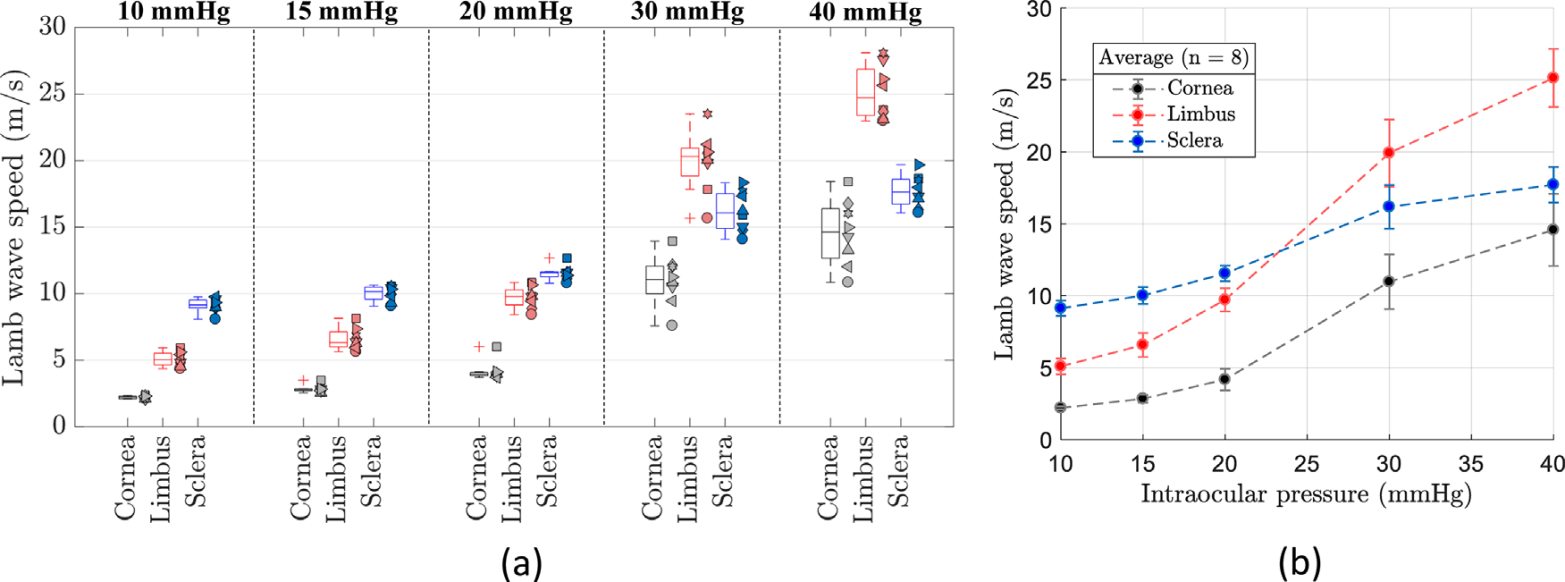
Statistical analysis of Lamb wave speed in all samples. (**a**) Lamb wave speed of cornea, limbus, and sclera for all eight eye samples as a function of IOP. Each sample is coded by the shape of the datapoints plotted alongside the corresponding box-and-whisker plot. The boxes are the interquartile range, the median is represented by the central line, the whiskers are the 5th and 95th percentiles, and crosses represents the outliers. (**b**) Average Lamb wave speed for all eye measurements (*n* = 8) versus IOP level. Error bars represent inter-sample standard deviation.

Lamb wave speed was averaged in the cornea, limbus, and sclera regions and plotted versus IOP in Figure 6 for every eye sample. Error bars in Figure 6 denote intra-region standard deviation of the wave speed. For all eye samples, Lamb wave speed progressively increased in each region with a different rate as the IOP increases. Interestingly, at IOPs of 20 and 30 mm Hg, the speed in the limbus surpassed the speed in the sclera, as revealed in Figure 5. Moreover, for the whole measured IOP range, the limbus was found to be highly nonlinear as measured by wave speed change (Δ*c_L_*∼18.5 m/s) compared to cornea (Δ*c_c_* ∼12.6 m/s) and sclera (Δ*c_s_*∼8.1 m/s). Figure 7a is a box plot of Lamb wave speed as a function of IOP of all (*n* = 8) eye samples. Here, each sample is coded by the shape of the datapoints plotted alongside the corresponding box-and-whisker plot. The boxes are the interquartile range, the median is represented by the central line, the whiskers are the 5th and 95th percentiles, and crosses represents the outliers. Finally, Figure 7b shows the average Lamb wave speed for all eye measurements (*n* = 8) versus IOP level. Error bars represent the inter-sample variability and confirm the extended compliance of the limbus compared to the cornea and sclera.

## Discussion

The first outcome of this work lies in the demonstration that elastic properties of the limbus can be inferred from wave propagation speed using OCE excited at 800 Hz and can be differentiated from the cornea and sclera when the eye-globe is subjected to deformation produced by a change in IOP. FEA predicted that, when propagating Lamb waves at 800 Hz, it is possible to measure changes in wave propagation speed in the limbus, even at a relatively low frequency of 800 Hz, when the Young's modulus of limbus was increased. Even though this can be observed in Figure 3h, the Lamb wave speed in limbus stops following the theoretical mRLFE model when its Young's modulus is greater than 300 kPa. This is most likely due to the wavelength becoming much longer than the lateral dimension of the limbus (∼ 1.4 mm) when its Young's modulus increased, leading to an underestimation of speed as demonstrated by Kirby et al.^13^ Similarly, the same effect was observed in the sclera (see Fig. 3i) when its Young's modulus was greater than 800 kPa. Therefore, this finite element analysis is importance to understand the limitations in accuracy of speed estimation using wave-based elastography of heterogeneous and shell-shaped tissues, such as the anterior eye.

The OCE experimental results demonstrated a different elasticity of the cornea, limbus, and sclera and a marked nonlinearity as a function of IOP. Moreover, the limbus speed variability (error bars in Fig. 6) is systematically larger compared to the corneal and sclera regions. This behavior is due to the fewer number of samples representing the small lateral extent of the limbal region, limiting the average wave speed calculation to ∼ 2 mm. For normal physiological IOP levels (10 to 15 mm Hg), we can back calculate the average speed values reported in Figure 7a into Young's moduli using the FEA plots of Figures 3g to 3i for each tissue case. We found $E_{c}∼24$ kPa, $E_{L}∼300$ kPa, and $E_{s}∼2.222$ MPa, for IOP = 10 mm Hg; and $E_{c}∼59$ kPa, $E_{L}∼810$ kPa, and $E_{s}∼3.396$ MPa, for IOP = 15 mm Hg. Young's moduli in the cornea and sclera are consistent with previous reported results.^30^^,^^41^^,^^42^ For higher IOPs, the back conversion is not possible because the curves in Figures 3g to 3i do not follow the mRLFE model in the sclera and limbus.

The limbal tissue was found to be highly nonlinear compared to the cornea and sclera, which suggests that the limbus has more biomechanical freedom to adjust its elasticity in response to global deformations, which were simulated by changes in IOP in this work. This is consistent with previous studies that suggested that the limbus has the structural flexibility to adjust for corneal deformations in order to maintain the visual acuity and still provide enough corneal support to avoid significant shape changes.^9^^–^^11^ It is of particular interest to compare this work with the numerical simulation predictions by Asejczyk-Widlicka, et al.,^10^ who provided optimal values of limbal Young's modulus to balance the moduli of the cornea and sclera for the preservation of vision quality. Figure 7b shows an increasing tendency of the limbal speed when the sclera/cornea speed ratio deceases, which is in agreement with predictions by Asejczyk-Widlicka et al.^10^ after the Young's modulus of the limbus reached a peak. Future work will focus on the simultaneous elastography of the anterior eye segment and evaluation of vision aberrations produced by IOP changes.

Understanding the anisotropic properties of the limbus is of great importance in the interpretation of results of this study. As indicated by Elsheikh et al.^12^ and other previous studies,^9^ the collagen fibril distribution in the limbus is arranged circumferentially, and studies in human subjects by Hjortdal^9^ demonstrated that Young's modulus measured along circumferential direction of the limbus are significantly higher not only than meridional measurements in limbus, but also along the central cornea. This suggests that the limbus is an anisotropic elastic tissue that will try to prevent changes in the ring diameter (circumferential deformations) when IOP is increased compared to deformations along the thickness. Therefore, relationships between Young's and shear moduli are not straightforward anymore as in the isotropic case. In our study, the Lamb wave speed measured by OCT is determined by the shear modulus along *xz*-plane without any assumption of the tissue anisotropy, and it can be converted into Young's modulus by assuming that the tissue under study is isotropic. On the other hand, reports by Elsheikh et al.^12^ and Hjortdal^9^ provide measurements of meridional Young's modulus (along the x-axis). Then, due to the anisotropic properties of the limbus, the direct comparison of Lamb wave speed in our study and meridional Young's modulus is not possible until further research on the specific type of anisotropic model of the limbus that can establish the relationships between Young's and shear moduli along the desired directions.

The limitations of this study should be mentioned. First, the FEA did not account for the elastic anisotropy and nonlinearity of the ocular tissues, which has been reported in the cornea and sclera.^43^^–^^46^ Therefore, the Lamb wave speed measurements would be more related to the out-of-plane shear modulus (along the depth axis) than the Young's modulus. On the other hand, OCE experiments were conducted at a single excitation frequency of 800 Hz, preventing the calculation of dispersion curves and the subsequent estimation of viscoelastic properties of the eye, as previously reported using transient techniques.^30^^,^^31^ The reasons behind this decision lay in the great difficulty of transient methods in generating smaller wavelengths (∼ 1 mm) with sufficient displacement amplitude capable of propagating along the whole field of view. Moreover, as shown in Figures 3g to 3i, the effect of different thicknesses in the cornea, limbus and sclera segments produce wave speed differences of < 1 m/s assuming the same Young's modulus for all regions. Even though such discrepancies are nontrivial, they are much smaller than the speed variations detected in this study when increasing IOP. In addition, eyeball samples showed some evidence of swelling, which may change the mechanical properties and thickness of the cornea and sclera. Our previous research found changes in the wave speed (Δ*c* < 0.5 m/s) in porcine corneas after an hour of hydration with PBS.^47^ Because our study is measuring larger variation of wave speed (Δ*c* > 8 m/s) in eyes exposed at the same level of hydration under variations of IOP, we could consider this effect to be of low impact when ocular tissues are measured individually. Nevertheless, swollen corneas may have a different biomechanical response than physiological hydrated corneas; therefore, results in Figure 7 could be different under in vivo conditions, which may be explored in our future work. Finally, this whole study has been conducted in ex vivo porcine eyeballs and a direct extrapolation of our results to human eyes may require considering the impact of physiological hydration and collagen fibril distribution in the human anterior segment.

## Conclusion

We have presented experimental evidence of the biomechanical behavior of the limbus during eye-globe deformation produced by an elevation of IOP using wave-based OCE. Moreover, finite element simulations predicted that it is possible to measure changes in elastic wave speed, at 800 Hz excitation, in the limbus when its Young's modulus was increased. Results from eight porcine anterior eye segments revealed that the wave speed in limbus (*c_L_* = 6.5 m/s) was in between cornea (*c_c_* = 2.9 m/s) and sclera (*c_s_* = 10.0 m/s) for physiological IOP level (15 mm Hg) and rapidly increased as the IOP level was elevated even surpassing the scleral wave speed when reaching a sclera/cornea speed ratio less than ∼ 2.5. Moreover, the limbus was found to be highly nonlinear as measured by changes in elastic wave speed, (Δ*c_L_*∼18.5 m/s), compared to the cornea (Δ*c_c_* ∼12.6 m/s) and sclera (Δ*c_s_*∼8.1 m/s). This may suggest that the limbus have enough structural flexibility to stabilize anterior eye shape during IOP changes.
